# Intraoperative Low-Dose Methadone for Pediatric Posterior Spinal Fusion: A Single-Center Retrospective Cohort Study

**DOI:** 10.3390/children13030400

**Published:** 2026-03-13

**Authors:** Roshni Cheema, Kristina Boyd, Mihaela Visoiu, Hsing-Hua Sylvia Lin, Scott E. Licata, Ruth Ressler, Vishali Veeramreddy, Shraddha Sriram, Selena Rashid, Senthilkumar Sadhasivam, Paul Hoffmann

**Affiliations:** 1 Department of Anesthesiology and Perioperative Medicine, UPMC Children’s Hospital of Pittsburgh, University of Pittsburgh, Pittsburgh, PA 15224, USA; visoium@upmc.edu (M.V.); licatase@chp.edu (S.E.L.); resslerr@upmc.edu (R.R.); rashids@upmc.edu (S.R.); hoffmannpj2@upmc.edu (P.H.); 2 Department of Anesthesia, University of Pittsburgh, Pittsburgh, PA 15260, USA; boydkl2@upmc.edu (K.B.); srirams4@upmc.edu (S.S.); 3 Department of Anesthesiology and Perioperative Medicine, Clinical and Translational Science Institute, University of Pittsburgh, Pittsburgh, PA 15224, USA; hsl26@pitt.edu; 4 School of Medicine, Imperial College London, London SW7 2AZ, UK; vishali.veeramreddy@esneft.nhs.uk

**Keywords:** posterior spinal fusion, methadone, postoperative analgesia, opioid consumption, adolescent idiopathic scoliosis, pediatric

## Abstract

**Highlights:**

**What are the main findings?**

• In a 339-patient retrospective pediatric posterior spinal fusion cohort, perioperative low-dose methadone achieved pain control comparable to standard regimens, with similar pain scores across POD 0–3.
• Opioid use was higher on POD 0 in the methadone group but not different on POD 1–3; hospital length of stay was unchanged.

**What are the implications of the main findings?**

• Low-dose methadone is a feasible multimodal adjunct for pediatric PSF, providing stable analgesia without prolonging recovery or hospitalization.
• Variation in dosing and timing likely affected opioid outcomes, supporting the need for prospective, protocolized ERAS-based studies.

**Abstract:**

**Background:** Posterior spinal fusion (PSF) for adolescent idiopathic scoliosis causes significant postoperative pain and high opioid requirements. Methadone, with dual μ- and κ-opioid agonism and NMDA antagonism, provides long-acting analgesia and may reduce perioperative opioid use. This study evaluated whether perioperative low-dose methadone (0.1 mg/kg) improves postoperative pain and opioid outcomes after pediatric PSF. **Methods:** In this single-center retrospective cohort study (January 2019–June 2023), pediatric patients <23 years old undergoing PSF were categorized by perioperative methadone exposure (intraoperative and/or postoperative) versus no methadone. The primary outcome was total postoperative opioid consumption (morphine milligram equivalents per kilogram, MME/kg) over postoperative days (POD) 0–3. Secondary outcomes were average daily pain scores and hospital length of stay (LOS). Inverse probability weighting (IPW) adjusted for age, sex, and protocol period. **Results:** A total of 339 patients (51% no methadone, 49% methadone; mean age 14.6 ± 2.5 years; 76% female) were analyzed. Methadone patients had longer anesthesia (392 vs. 372 min, *p* = 0.042) and surgery times (287 vs. 266 min, *p* = 0.01). IPW-adjusted associations show postoperative opioid use was significantly higher in the methadone group on POD 0 (median 2.5 vs. 2.1 MME/kg in no methadone group; *p* = 0.005). No significant differences were found in postoperative average pain scores (e.g., mean NRS: 2.3 vs. 2.5 on POD 0, *p* = 0.12) and LOS (3.3 vs. 3.1 days, *p* = 0.38) between methadone group and no methadone group. **Discussion:** Perioperative methadone provided similar analgesia for pain management and recovery without prolonging hospitalization, despite higher early opioid use on POD 0. Retrospective design limits causal inference, and residual confounding may persist despite propensity score-based adjustments. Further prospective trials are required to establish safety and dosing. **Conclusions:** In this retrospective cohort, perioperative low-dose methadone was associated with higher early postoperative opioid use but no significant differences in pain scores or length of stay compared with standard regimens. Methadone did not demonstrate an opioid-sparing effect in this real-world setting. Prospective studies are needed to better define its role and safety in pediatric posterior spinal fusion.

## 1. Introduction

Adolescent idiopathic scoliosis (AIS) is among the most common spinal deformities in children [1,2], with a prevalence between 0.5% and 5% [3,4,5]. Surgical intervention, most often posterior spinal fusion (PSF), is indicated in patients with progressive curves, typically those exceeding 40 degrees [6], to prevent further spinal deformity, respiratory compromise, and functional disability. However, this surgery is associated with substantial postoperative pain due to extensive soft-tissue and bony dissection [6,7]. Effective pain control is critical to enhance recovery, reduce chronic postsurgical pain, and mitigate long-term opioid dependence [8,9,10,11].

Conventional pain management relies heavily on intravenous opioids via patient-controlled analgesia (PCA), which are associated with nausea, vomiting, respiratory depression, and delayed recovery [12,13,14,15,16]. Multimodal approaches combining opioids with adjuncts such as acetaminophen, NSAIDs, and muscle relaxants have improved outcomes, yet opioid-related side effects remain significant [17,18,19,20].

Methadone, a synthetic opioid with μ- and κ-receptor agonism, NMDA receptor antagonism, and monoamine reuptake inhibition [21,22,23], offers long-lasting analgesia from a single dose. Its extended half-life (24–36 h) may sustain postoperative pain relief and reduce opioid requirements [24,25]. Low-dose methadone maintains plasma levels below those associated with QTc prolongation or respiratory depression, supporting its safety in perioperative use [26]. Prior studies in adults and limited pediatric data suggest intraoperative methadone may reduce postoperative opioid consumption and stabilize analgesia [27]. However, pediatric data in real-world clinical settings remain limited.

This retrospective study evaluates the association of perioperative low-dose methadone with postoperative pain control, opioid consumption, and recovery outcomes in pediatric and adolescent patients undergoing PSF.

## 2. Materials and Methods

### 2.1. Study Population

This retrospective cohort study, approved by the Institutional Review Board of the University of Pittsburgh, PA, USA (Study # 20050148), used electronic health record (EHR) data [28] from pediatric patients who underwent spinal fusion at UPMC Children’s Hospital of Pittsburgh between January 2019 and June 2023. Eligible patients included pediatric and adolescent patients under 23 years of age, undergoing pediatric spinal fusion surgery (PSF) for idiopathic or adolescent scoliosis (AIS). Exclusions were missing exposure or outcome data, non-idiopathic scoliosis, revision surgery, pre-existing chronic pain diagnoses, opioid dependence, or incomplete procedural data. The upper age threshold of <23 years reflects institutional practice, as adolescent and young adult scoliosis patients up to age 23 are routinely managed within pediatric surgical and anesthesia pathways using the same PSF and perioperative analgesia protocols. Institutional Review Board approval was obtained (STUDY20050148).

### 2.2. Treatment Groups

Patients were classified as the methadone group if they received methadone intraoperatively and/or postoperatively during the index hospitalization. Those without any intraoperative or post-operative methadone exposure comprised the no methadone group. Methadone adoption followed a perioperative pain management protocol shift on 31 August 2021, introducing intraoperative methadone as a preferred analgesic option.

All patients received a standardized general anesthetic protocol, which included maintenance with Total intravenous anesthesia (TIVA) and opioid-based analgesia (e.g., remifentanil, fentanyl, or sufentanil). In the methadone group, patients who followed the protocol intraoperatively received two intravenous doses of methadone, 4 h apart (0.1 mg/kg and 0.05 mg/kg), followed by oral methadone (0.1 mg/kg) every 12 h on post-op day (PODs) 1 and 2. In both groups, postoperative pain management followed institutional Enhanced Recovery After Surgery (ERAS) protocols, including the use of patient-controlled analgesia (PCA) with either morphine or hydromorphone, acetaminophen, NSAIDs as tolerated, and adjunctive agents such as muscle relaxants.

Demographic data, surgical details, and perioperative medication administration were extracted from electronic medical records.

### 2.3. Outcome Measures

The primary outcome was daily postoperative opioid use (MME/kg) [28] over postoperative days (PODs) 0–3. Pain scores were aggregated from the FLACC, numeric rating scale (NRS), and visual analog scale (VAS) [28,29,30,31]. The secondary outcome was hospital length of stay (LOS).

### 2.4. Statistical Analysis

Descriptive statistics were calculated as means (standard deviations, SD) for normally distributed continuous variables, medians (interquartile range, IQR) for skewed data, and counts (percentages) for categorical data. Group differences were assessed with Student’s *t*-tests or Wilcoxon rank-sum tests [28] for continuous data and χ^2^ or Fisher’s exact tests for categorical variables.

To balance observed and potential confounding and to approximate a randomized controlled trial, inverse probability weighting (IPW) was used [28]. IPW was derived from propensity scores. Propensity score models were fit using logistic regression with the methadone treatment group as the outcome, and age, sex, and protocol time period (defined by pre/post windows of standard operating protocol, shifted on 31 August 2021) as predictors.

Diagnostics of histograms and measures of spread were analyzed to ensure common support of IPW between treatment groups. Standardized mean differences (SMDs) for demographics between treatment groups were reviewed before and after IPW, with SMDs < 0.2 indicating balance [28]. Supplemental Table S1 presents the confounding variables included in the propensity score model and their corresponding SMDs pre- and post-IPW.

Outcome summary statistics [28] were calculated using unadjusted and IPW-adjusted data. For our primary outcomes of MME/kg and pain scores, IPW-adjusted longitudinal linear mixed models were fit to analyze between-methadone-group differences over time. Random effects were fit per patient, fixed effects for time and treatment group. Time points included were postoperative days (POD) 0–3. Interaction terms were tested and included if significant. If significant, daily contrasts were calculated to test for IPW and within-person adjusted between-group differences. Interaction plots were generated from interaction models to visualize model estimates. Our secondary outcomes of hospital LOS and overall POD 1–3 post-operative MME per kg were modeled using log gamma IPW regression models.

All analyses were conducted using R version 4.3.3 (R Core Team, 2024). Missing values were excluded from denominators and statistical tests. Statistical significance was defined as *p* < 0.05. Propensity scores and inverse probability weights were estimated using the WeightIt package. Weighted descriptive statistics and generalized linear models were fit with the survey package. Linear mixed-effects models incorporating IPW were fit using lmerTest, with random intercepts for patients and fixed effects for treatment group, postoperative day, surgical duration in minutes, and intraoperative opioid consumption measured in MME per kg.

An additional adjusted analysis separated methadone exposure into three groups (no methadone, intraoperative methadone only, and intraoperative plus postoperative methadone). Multivariable models adjusted for age, sex, surgical duration, and protocol era to further account for operative complexity and temporal practice changes.

Because of the retrospective design, no power calculation was performed a priori before the study, and sample size was determined by the number of eligible cases available during the study period which is large enough to have adequate statistical power with some significant difference.

A prespecified sensitivity analysis was performed restricting the cohort to patients <18 years of age to evaluate whether inclusion of older adolescents and young adults influenced primary outcomes.

## 3. Results

### 3.1. Baseline Characteristics and Treatment Groups

The final analytic cohort included 339 patients, with 173 (51%) in the no methadone group and 166 (49%) in the methadone group (Table 1). The mean age was 14.6 years (SD 2.5 years), and 76% were female. Groups were similar in age, sex, and weight. Patients in the methadone group had significantly longer anesthesia (392 vs. 372 min, *p* = 0.042) and surgery times (287 vs. 266 min, *p* = 0.014). Surgical timeframe differed substantially: nearly half of methadone patients were in the post-protocol era compared to 27% of non-methadone patients (*p* < 0.001) (Table 1). Intraoperative and postoperative opioid exposure can be seen in Table 2a,b, while Table 2c highlights methadone administration in intraoperative and postoperative periods. In a sensitivity analysis limited to patients <18 years (n = 300), outcome patterns were unchanged compared with the full cohort.

### 3.2. Primary Outcomes—Postoperative Opioid Use in MME/kg and Postoperative Pain Scores

**Figure 1:**
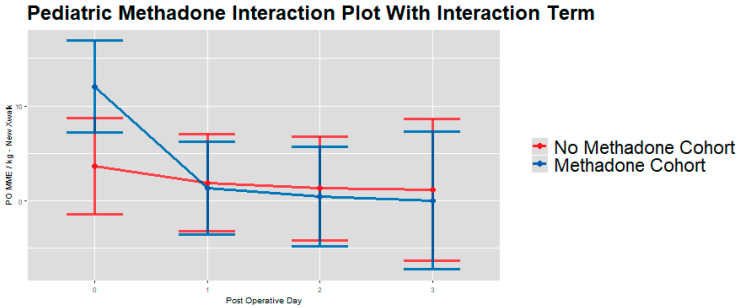
IPW adjusted linear mixed model interaction plot showing post-operative MME/kg [28] (y-axis) for whole sample cohort on PODs 0–3 (x-axis), by methadone group. Estimates showing model-estimated means and confidence intervals around them. Interaction terms are included in estimates. *p*-values of group differences in each POD are: POD 0 = 0.0185, POD 1 = 0.873, POD 2 = 0.822, and POD 3 = 0.830. We observe no significant between-group differences by day on PODs 1–3.

Unadjusted analysis showed our primary outcome—total postoperative opioid use across PODs 0–3 in MME/kg [28]—to be significantly lower in the no methadone group compared to the methadone group (median = 3.5 vs. 4.3; *p* = 0.003). This difference was primarily driven by higher opioid consumption in the methadone group on POD 0 (2.1 vs. 2.5; *p* = 0.005). Opioid use on PODs 1–3 [28] was similar between treatment groups (Table 3). IPW-adjusted medians demonstrated the same trends in opioid consumption (Table 4). IPW-adjusted linear mixed models [28] showed patients in the methadone group had higher postoperative opioid use (mean ratio 1.66, 95% CI: 1.05–2.62; *p* = 0.03, Table 5) compared to those in the no methadone group over PODs 0–3 [28]. There was a significant interaction between POD and methadone group [28]; adjusted contrasts by day are shown in Figure 1, with between-group differences significant on POD 0 (*p* = 0.0185) but not on PODs 1–3.

**Figure 2:**
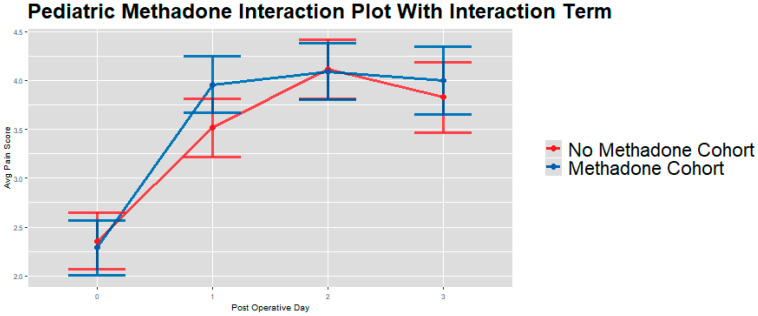
IPW adjusted linear mixed model interaction plot showing average pain scores (y-axis) for whole sample cohort on PODs 0–3 (x-axis), by methadone group [28]. Estimates showing model-estimated means and confidence intervals around them. Interaction terms are included in estimates. *p*-values of group differences in each POD are: POD 1 = 0.036, POD 2 = 0.905, and POD 3 = 0.497. We observe no significant between-group differences by day on PODs 2–3 and significant between-group differences on POD 1.

Unadjusted analyses, IPW-adjusted univariate group comparisons, and IPW-adjusted linear mixed models showed no consistent or significant differences in either maximum or average pain scores across PODs 0–3 (Table 3, Table 4 and Table 5). The only exception was a modest difference in average pain scores on POD 1 (*p* = 0.036, Figure 2), which was not sustained on PODs 2–3.

Sensitivity analyses restricted to patients <18 years showed similar findings, with no clinically meaningful between-group differences in postoperative pain scores and persistently but modestly higher opioid use in the methadone group.

### 3.3. Three Group Methadone Exposure Analysis

In adjusted three-group models (no methadone, intraoperative methadone only, intraoperative plus postoperative methadone), there were no clinically or statistically meaningful differences in postoperative pain scores or hospital length of stay. Intraoperative methadone only group was not associated with reduced postoperative opioid consumption compared with no methadone group (MME ratio 0.97, *p* = 0.92). Patients who received both intraoperative and postoperative methadone had substantially higher postoperative opioid exposure (~3.6-fold vs. both other groups, *p* < 0.001), driven primarily by POD0 administration. This is possibly due to a practice variation with the pain team managing postoperative pain including use of postoperative methadone. The differences persisted after adjustment for age, sex, surgical duration, and protocol era.

### 3.4. Secondary Outcomes—LOS and Overall Post-Operative MME Consumption

**Figure 3:**
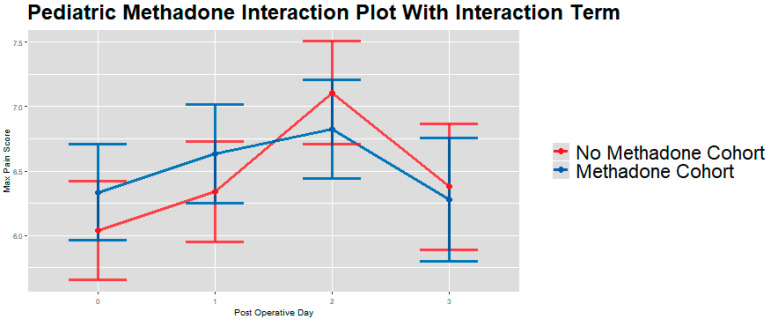
IPW adjusted linear mixed model interaction plot showing maximum pain scores (y-axis) for whole sample cohort on PODs 0–3 (x-axis), by methadone group [28]. Estimates showing model-estimated means and confidence intervals around them. Interaction terms are included in estimates. *p*-values of group differences in each POD are: POD 1 = 0.286, POD 2 = 0.313, and POD 3 = 0.772. We observe no significant between-group differences by day on PODs 1–3.

When pooled across PODs 0–3, IPW-adjusted linear mixed models [28] showed that patients in the methadone group had significantly higher overall postoperative opioid consumption by weight compared to the no methadone group (mean ratio 1.66, 95% CI: 1.05–2.62; adjusted mean difference 8.47 MME/kg, 95% CI: 1.42–15.51; *p* = 0.019; Table 5). These results indicate that, after weighing baseline covariates, methadone exposure was associated with greater cumulative opioid use across the early postoperative period, even though day-specific differences were largely limited to POD 0 (Figure 1, Figure 2 and Figure 3).

By contrast, unadjusted analyses, IPW-adjusted univariate group comparisons, and IPW-adjusted gamma regression models showed no significant differences in postoperative hospital LOS between groups (median 3.1 vs. 3.3 days; *p* = 0.376; Table 3, Table 4 and Table 5).

## 4. Discussion

In this large single-center retrospective cohort, perioperative low-dose methadone use in pediatric posterior spinal fusion was associated with higher early postoperative opioid consumption, but similar pain scores and hospital length of stay compared to traditional regimens. These findings suggest that while methadone did not demonstrate an opioid-sparing effect in this heterogeneous clinical practice setting, it provided similar analgesic efficacy and recovery without prolonging hospitalization.

Several factors likely influenced the observed trends. The pharmacologic profile of methadone may also help explain the observed temporal pattern of opioid use. Methadone has a delayed peak analgesic effect (approximately 4–8 h) and a prolonged duration of action of roughly 24–36 h. In our cohort, methadone was administered intraoperatively rather than preoperatively; therefore, immediate post-extubation pain on POD 0 would still be expected to require supplemental short-acting opioids. The similar pain scores observed on PODs 1–3 despite higher early opioid use are consistent with a delayed and sustained analgesic contribution from methadone in the later postoperative period. The introduction of methadone coincided with an institutional transition to a formalized pain management protocol and increased involvement of a dedicated pediatric pain service. This shift may have led to more proactive opioid administration and documentation, particularly during the early postoperative phase (POD 0). Additionally, longer anesthesia and surgical times in the methadone cohort may reflect more complex cases or differing surgical teams—factors independently associated with increased postoperative analgesic needs. Moreover, methadone dosing was not standardized across all patients in the methadone group, who were enrolled before the introduction of the protocol.

Additional exposure-stratified analyses further clarified these findings. When methadone use was separated into intraoperative-only versus intraoperative plus postoperative administration, intraoperative methadone alone was not associated with opioid-sparing or improved analgesia compared with no methadone. The markedly higher opioid exposure observed in patients receiving postoperative methadone likely reflects confounding by indication, with methadone preferentially administered to patients with greater anticipated or observed analgesic needs rather than producing improved outcomes. This could also be due to clinical practice variation with involvement of acute pain team using postoperative methadone and other opioids more liberally than surgeons managing postoperative pain. Larger prospective studies comparing these three groups with standardized surgical and anesthesia care are needed to overcome the limitations of this retrospective study.

Importantly, the similarity in pain scores despite these confounding factors suggests that methadone provided stable analgesia and did not result in inadequate pain control. The absence of prolonged recovery or increased length of stay (LOS) reinforces methadone’s safety and clinical viability as part of multimodal pediatric analgesia. These results align with prior pediatric reports demonstrating the feasibility of low-dose methadone protocols without respiratory or cardiac complications, and with adult literature supporting methadone’s potential to reduce opioid variability and stabilize analgesia duration.

Mok et al., in a retrospective study, demonstrated that a protocol utilizing intraoperative and scheduled postoperative methadone doses resulted in a 45% reduction in opioid use compared to a patient-controlled analgesia (PCA)-based protocol, while achieving similar analgesia after pediatric posterior spinal fusion [27].

More recently, a 2023 double-blind randomized controlled trial by Fons et al. concluded that a two-dose intraoperative methadone regimen significantly reduced opioid consumption compared to morphine, while maintaining comparable pain scores between groups [32].

The pharmacologic rationale for methadone—long half-life, NMDA antagonism, and monoaminergic modulation—remains compelling for sustained pain control and reduced central sensitization. Even though our retrospective data did not show reduced opioid consumption, methadone may still offer advantages by preventing breakthrough pain and smoothing the postoperative analgesic trajectory. We were unable to reliably evaluate PONV outcomes in this retrospective dataset, but this remains an important patient-centered endpoint for future prospective methadone studies. Prospective trials are warranted to optimize dosing, timing (intra- versus postoperative), and integration within enhanced recovery protocols.

The findings of this study help define priorities for future investigation. Prospective studies should evaluate standardized methadone dosing and timing strategies, including pre-incision versus intraoperative administration, protocolized multimodal co-analgesic regimens, and predefined patient-centered outcomes such as recovery quality, opioid-related side effects, and functional recovery. Randomized or protocol-driven comparative studies within enhanced recovery pathways will be particularly important to determine whether methadone’s pharmacologic advantages translate into consistent opioid-sparing and recovery benefits in pediatric spinal fusion populations.

## 5. Limitations

This study’s retrospective design limits causal inference and is subject to residual confounding. Institutional transitions, variable surgical complexity, surgeons and durations of surgery, and non-standardized methadone dosing especially preferential use of methadone in more invasive and longer procedures may have influenced results. Our statistical approach (IPW + mixed models + duration covariates) partially addresses case complexity including the length of surgery but does not fully replace detailed surgical severity metrics like Cobb angle, levels and laminectomies. Postoperative methadone administration overlapped with the intraoperative protocol period, complicating exposure classification. Postoperative nausea and vomiting (PONV) were not analyzed, as it was not a predefined endpoint and retrospective documentation of nausea and vomiting symptoms and antiemetic use were not sufficiently standardized for reliable comparison. PONV is a multifactorial outcome influenced by patient risk factors, anesthetic technique, opioid exposure, and prophylaxis strategies, and will be better evaluated in prospective, protocolized analyses. Additionally, longer-term outcomes such as chronic pain or persistent opioid use were not evaluated.

## 6. Conclusions

Intraoperative low-dose methadone was associated with similar postoperative pain scores, but higher early opioid consumption compared to standard care. However, opioid requirements equalized by POD 1–3, likely due to methadone’s long half-life and sustained analgesic effect. Hospital length of stay was comparable between groups, indicating methadone did not delay recovery. However, systematic assessment of adverse events was not performed, and safety conclusions require prospective evaluation.

## Figures and Tables

**Table 1:** Baseline characteristics by methadone analgesia groups [28].

Variable [28]	Variable Value	Total n = 339	No Methadone n = 173 (51%)	Methadone n = 166 (49%)	*p*-Value
Age, mean (SD)	years	14.6 (2.51)	14.5 (2.5)	14.7 (2.6)	0.482
Sex, n (%)	Female	259 (76.4)	130 (75)	129 (78)	0.578
	Male	80 (23.6)	43 (25)	37 (22)	
Weight, mean (SD)	kg	56.44 (17.15)	55.8 (17.2)	57.1 (17.2)	0.559
Anesthesia duration, mean (SD)	Minutes	382.11 (82.65)	372.4 (87.5)	392.2 (76.2)	0.042
Surgery duration, mean (SD)	Minutes	276.01 (72.74)	265.9 (74.5)	286.5 (69.5)	0.014
Scheduled Procedure, n (%)	Spinal Fusion Posterior (M-Orthopedic)	314 (92.6)	158 (91)	156 (94)	0.410
	Other	25 (7.4)	15 (9)	10 (6)	
Surgical Timeframe, n (%)	Post-protocol change	126 (37.2)	47 (27)	79 (48)	<0.001
	Pre-protocol change	213 (62.8)	126 (73)	87 (52)	

**Table 2:** (**a**) Intraoperative opioid medications by methadone analgesia groups. (**b**) Post-operative opioid medications by methadone analgesia groups. (**c**) Methadone exposure (MME/kg) by period.

(**a**)				
**Variable**	**Total** **n = 339**	**No Methadone** **n = 173 (51%)**	**Methadone** **n = 166 (49%)**	***p*** **-Value**
Intraoperative Opioid MME/kg, Median (IQR)	17.2 (3.74–24.3)	16.3 (5.8–23.1)	18.6 (0.5–24.5)	0.464
Intraoperative Opioid MME/kg, mean (SD)	15.45 (11.64)	15.6 (10.6)	15.3 (12.7)	0.833
Received Fentanyl, n (%)	209 (61.65)	132 (77.2)	77 (46.4)	<0.001
Received Methadone, n (%)	166 (48.96)	0 (0)	166 (100)	<0.001
Received Morphine, n (%)	172 (50.7)	135 (78.9)	37 (22.3)	<0.001
Received Remifentanil, n (%)	220 (64.89)	115 (67.3)	105 (63.3)	0.097
Received Sufentanil, n (%)	47 (13.86)	41 (24.0)	6 (3.6)	<0.001
(**b**)				
**Variable**	**Total** **n = 339**	**No Methadone** **n = 173 (51%)**	**Methadone** **n = 166 (49%)**	***p*** **-Value**
Postoperative Opioid MME/kg, Median (IQR) [28]	3.77 (2.47–6.04)	3.5 (2.3–5.2)	4.3 (2.5–7.5)	<0.002
Postoperative Opioid MME/kg, mean (SD)	5.22 (55.51)	1.4 (1.5)	9.1 (78.9)	0.206
Received Fentanyl, n (%)	89 (26.25)	42 (24.6)	47 (28.3)	0.399
Received Methadone, n (%)	43 (12.7)	0 (0)	43 (26)	<0.001
Received Morphine, n (%)	320 (94.39)	159 (93)	161 (97)	0.130
Received Oxycodone, n (%)	316 (93.21)	158 (92.4)	158 (95.2)	0.303
Received Remifentanil, n (%)	1 (0.2)	0 (0)	1 (0.6)	0.307
(**c**)				
**Period**	**n Patients**		**Mean (SD)**	**Methadone (IQR)**
Intraoperative	166		0.09 (0.04	0.09 (0.06–0.12)
Postoperative	43		0.26 (0.06)	0.28 (0.21–0.30)

**Table 3:** Unadjusted associations between methadone groups and outcomes.

Outcome Variable	Total n = 339	No Methadone n = 173 (51%)	Methadone n = 166 (49%)	*p*-Value
**Postoperative Opioid Use in MME/kg, mean (SD)**				
Total Postoperative Opioid Use in MME/kg across POD 0–3	8.96 (56.0)	4.51 (5.58)	13.5 (79.4)	0.146
POD 0	7.24 (56.33)	2.79 (4.75)	11.72 (79.52)	0.003
POD 1	1.22 (1.96)	1.14 (1.64)	1.29 (2.24)	0.278
POD 2	0.51 (0.34)	0.54 (0.39)	0.48 (0.28)	0.284
POD 3	0.46 (0.29)	0.47 (0.23)	0.46 (0.35)	0.269524
**Postoperative Opioid Use in MME/kg, median (IQR)**				
POD 0	2.29 (1.39–3.44)	2.10 (1.33–2.87)	2.49 (1.43–4.95)	0.003
POD 1	0.89 (0.49–1.40)	0.84 (0.43–1.29)	0.92 (0.55–1.40)	0.278
POD 2	0.45 (0.30–0.61)	0.46 (0.30–0.69)	0.45 (0.30–0.60)	0.284
POD 3	0.44 (0.26–0.57)	0.45 (0.28–0.59)	0.43 (0.25–0.56)	0.269
**Average Pain Score, mean (SD)**				
POD 0	2.4 (1.58)	2.5 (1.7)	2.3 (1.5)	0.386
POD 1	3.85 (1.93)	3.7 (1.8)	4 (2)	0.069
POD 2	4.21 (2.01)	4.3 (1.8)	4.2 (2.2)	0.650
POD 3	4.24 (2.13)	4.2 (2.1)	4.3 (2.2)	0.497
**Max Pain Score, mean (SD)**				
POD 0	6.3 (2.58)	6.2 (2.6)	6.4 (2.6)	
POD 1	6.67 (2.38)	6.6 (2.4)	6.8 (2.4)	0.356
POD 2	7.13 (2.46)	7.3 (2.3)	6.9 (2.6)	0.183
POD 3	6.68 (2.56)	6.8 (2.4)	6.6 (2.7)	0.833
**Postoperative Hospital LOS, median (IQR)**	3.2 (2.88–3.9)	3.1 (2.8–3.9)	3.5 (2.9–3.9)	0.345

Abbreviations: POD, postoperative day; kg, kilogram; MME, morphine milligram equivalents; LOS = length of stay [28].

**Table 4:** IPW adjusted associations between methadone groups and outcomes.

Outcome Variable	Total n = 678	No Methadone n = 338.96 (50.0%)	Methadone n = 339.04 (50.0%)	*p*-Value
**Postoperative Opioid Use in MME/ kg, mean (SD)**				
Total Post-Operative Opioid Use in MME/kg POD 0–3	9.24 (3.32)	4.51 (0.59)	13.88 (9.26)	0.153
POD 0	7.49 (58.50)	2.77 (4.69)	12.10 (81.91)	0.158
POD 1	1.21 (1.90)	1.14 (1.59)	1.28 (2.17)	0.512
POD 2	0.51 (0.34)	0.54 (0.39)	0.48 (0.28)	0.124
POD 3	0.46 (0.29)	0.47 (0.23)	0.46 (0.24)	0.821
**Postoperative Opioid Use in MME/ kg, median (IQR)**				
POD 0	2.28 (1.39–3.43)	2.1 (1.4–2.9)	2.5 (1.4–4.7)	0.005
POD 1	0.89 (0.49–1.40)	0.9 (0.4–1.4)	0.9 (0.6–1.5)	0.335
POD 2	0.45 (0.30–0.62)	0.5 (0.3–0.7)	0.4 (0.3–0.6)	0.283
POD 3	0.44 (0.26–0.57)	0.5 (0.3–0.6)	0.4 (0.3–0.6)	0.214
**Average Pain Score, mean (SD)**				
POD 0	2.39 (1.58)	2.51 (1.66)	2.28 (1.49)	0.176
POD 1	3.85 (1.94)	3.68 (1.85)	4.02 (2.03)	0.110
POD 2	4.20 (2.01)	4.27 (1.82)	4.14 (2.18)	0.542
POD 3	4.23 (2.13)	4.16 (2.09)	4.30 (2.18)	0.644
**Max Pain Score, mean (SD)**				
POD 0	6.29 (2.58)	6.26 (2.59)	6.33 (2.58)	0.804
POD 1	6.67 (2.39)	6.58 (2.37)	6.75 (2.42)	0.532
POD 2	7.12 (2.47)	7.35 (2.28)	6.90 (2.63)	0.101
POD 3	6.68 (2.57)	6.78 (2.43)	6.57 (2.71)	0.588
**Postoperative Hospital LOS, median (IQR)**	3.2 (2.9–3.9)	3.1 (2.8–3.9)	3.3 (2.9–3.9)	0.376

Abbreviations: IPW, inverse probability weighted; POD, postoperative day; kg, kilogram; MME, morphine milligram equivalents; LOS = length of stay [28].

**Table 5:** IPW adjusted associations between methadone group vs. no methadone group (reference) and clinical outcomes over postoperative days 0–3.

Outcomes	Pooled PO Opioid Use over POD 0–3	PO Opioid Use in MME/kg	Average Pain Score	Max Pain Score	Postoperative Hospital LOS
**Estimates**	Mean Ratio 95% CI	Mean Difference 95% CI	Mean Difference 95% CI	Mean Difference 95% CI	Mean Ratio 95% CI
**Overall (n = 678.00)**	*p* = 0.03 1.66 (1.05, 2.62)	*p* = 0.019 8.47 (1.42, 15.51)	*p* = 0.74 −0.07 (−0.47, 0.34)	*p* = 0.27 0.3 (−0.24, 0.83)	*p* = 0.69 0.99 (0.93, 1.05)

Abbreviations: kg, kilogram; MME, morphine milligram equivalents; LOS, length of stay; PO, post-operative [28].

## Data Availability

The original contributions presented in this study are included in the article. Further inquiries can be directed to the corresponding author.

## Supplementary Materials

The following supporting information can be downloaded at: https://www.mdpi.com/article/10.3390/children13030400/s1, Table S1: Propensity Score Covariate Balancing.
