# Supplementary material for: Intraoperative Low-Dose Methadone for Pediatric Posterior Spinal Fusion: A Single-Center Retrospective Cohort Study
Source: Children (Basel). 2026 Mar 13;13(3):400. doi: 10.3390/children13030400 (PMC13025091; doi:10.3390/children13030400)
Supplement: Supplementary file 1 [file children-13-00400-s001.zip › children-4143650-supplementary.pdf]

Supplemental Table S1. Propensity Score Covariate Balancing.

Propensity Score Model Predictor	Unweighted SMD	IPW SMD
Age	0.086	0
Sex	0.067	0
Scheduled Procedure	0.07	0.094
Surgical Treatment Time Frame	0.391	0.452

Displaying between treatment group standardized mean differences (SMD) for each predictor used in the propensity score modeling. We chose SMD <= 0.2 as our cutoff for balanced covariates. All covariates except surgical treatment timeframe showed SMDs below our threshold post-weighting.
Abbreviations: SMD, standardized mean differences; IPW, inverse probability weighting
